# Feature Fusion of ICP-AES, UV-Vis and FT-MIR for Origin Traceability of *Boletus edulis* Mushrooms in Combination with Chemometrics

**DOI:** 10.3390/s18010241

**Published:** 2018-01-15

**Authors:** Luming Qi, Honggao Liu, Jieqing Li, Tao Li, Yuanzhong Wang

**Affiliations:** 1 Institute of Medicinal Plants, Yunnan Academy of Agricultural Sciences, Kunming 650200, China; 18669326801@163.com; 2 State Key Laboratory Breeding Base of Systematic Research, Development and Utilization of Chinese Medicine Resources, Chengdu University of Traditional Chinese Medicine, Chengdu 611137, China; 3 College of Agronomy and Biotechnology, Yunnan Agricultural University, Kunming 650201, China; honggaoliu@126.com (H.L.); lijieqing2008@126.com (J.L.); 4 College of Resources and Environment, Yuxi Normal University, Yuxi 653100, China

**Keywords:** origin traceability, *Boletus edulis*, ICP-AES, UV-Vis, FT-MIR

## Abstract

Origin traceability is an important step to control the nutritional and pharmacological quality of food products. *Boletus edulis* mushroom is a well-known food resource in the world. Its nutritional and medicinal properties are drastically varied depending on geographical origins. In this study, three sensor systems (inductively coupled plasma atomic emission spectrophotometer (ICP-AES), ultraviolet-visible (UV-Vis) and Fourier transform mid-infrared spectroscopy (FT-MIR)) were applied for the origin traceability of 184 mushroom samples (caps and stipes) in combination with chemometrics. The difference between cap and stipe was clearly illustrated based on a single sensor technique, respectively. Feature variables from three instruments were used for origin traceability. Two supervised classification methods, partial least square discriminant analysis (FLS-DA) and grid search support vector machine (GS-SVM), were applied to develop mathematical models. Two steps (internal cross-validation and external prediction for unknown samples) were used to evaluate the performance of a classification model. The result is satisfactory with high accuracies ranging from 90.625% to 100%. These models also have an excellent generalization ability with the optimal parameters. Based on the combination of three sensory systems, our study provides a multi-sensory and comprehensive origin traceability of *B. edulis* mushrooms.

## 1. Introduction

In many countries, specific regulations have been introduced to protect the geographical indications of agricultural products and foodstuffs in order to satisfy consumer requirements. Edible mushrooms are one of the most popular food resources that are very appreciated by consumers due to their aromatic flavor, and abundant nutritional and medicinal properties. As widely-consumed food products, their origin traceability is also an important step for assuring quality and safety. Firstly, their chemical constitution and corresponding nutritional and pharmacological functions obviously vary depending on geographical origins [1,2,3]. There is another reason for discriminating based on geographical origins. The accumulation of many beneficial and hazardous mineral elements is obviously different between different geographical origins [4,5].

*Boletus edulis* Bull, belonging to *Boletus,* is one of the most well-known edible mushrooms in Europe, North America and Asia. Compared with other mushrooms, this species has higher nutritional and pharmacological values [6,7,8]. Usually, it is appreciated as a flavorsome and delicious food material in cooking and, for example, it has a remarkable importance in the Italian culinary tradition [9]. Hence, many modern studies are focusing on them, demonstrating that their fruiting bodies are rich in many beneficial constituents such as proteins, polysaccharides, minerals, amino acids [10,11]. They have also been proved to show many pharmacological activities such as antioxidant, antibacterial and anti-inflammatory [12]. These highlighted properties lead these mushrooms to be widely consumed food products in the world. However, consumer acceptance mostly depends on their quality and safety, which is obviously related to geographical origins.

To date, various analytical approaches have been implemented to determine important components or metabolic fingerprints to analyze the quality of plants and food [13,14,15,16]. Many sensor systems have also been well conducted for the discrimination of mushrooms according to different microhabitats. Although these chemical profiles are unique and have been demonstrated to be promising, they are separately applied and insufficient to obtain comprehensive metabolic information. Besides, compared with other factors, geographical characterization is not an easy classification problem, as it is determined by many complicated and interactional ecological factors such as temperature, rainfall, elevation and so on. Therefore, a single sensor system will be subjected some new challenges for comprehensive and accurate origin traceability when more factors are involved.

Comparatively, data fusion techniques may provide an alternative strategy to enhance the accuracy and completeness for the geographical characterization of mushrooms [17]. This strategy can combine the chemical information from several sensor systems and, therefore, provide a deep and comprehensive perspective for understanding complex data variables. However, simple combination may be not optimal because the fused data matrix is too large and many redundant variables may be added. Therefore, many feature extraction procedures such as principle component analysis (PCA), partial least square discriminant analysis (PLS-DA) and other techniques have always been applied to screen effective characteristics in advance. It has been successfully applied to detect the quality of many commodities such as wines [18], foods [19] and medicinal plants [20]. These studies have demonstrated that data fusion on the feature level may present more effective and accurate chemometric characterization for different samples.

In view of the necessity of origin traceability and the superiority of feature fusion, three sensor techniques of inductively coupled plasma atomic emission spectrophotometer (ICP-AES), ultraviolet-visible (UV-Vis) and Fourier transform mid-infrared (FT-MIR) were jointly applied to collect the chemical information and conduct the origin traceability of *B. edulis* mushrooms, in combination with chemometrics. This work can provide a reference for the quality control of this mushroom species.

## 2. Materials and Methods

### 2.1. Sample Preparation

Eleven microhabitats were chosen to represent the wide distribution of *B. edulis* mushrooms in Yunnan Province (Figure S1). Well-developed mushroom individuals were collected between June and September from each microhabitat. Firstly, they were cleaned and washed with deionized water. Each fruiting body (Figure S2) was divided into cap and stipe in order to characterize the difference between two parts. Then these samples were dried in a drying oven at 60 °C to constant weight. Dried samples were smashed by a pulverizer and a sample powder was passed through a 100 mesh sieve. Finally, the obtained powder was stored in a dry condition. Cap and stipe were separately tested and a total of 184 samples were collected. The detailed information is shown in Table 1.

### 2.2. Instruments and Reagents

A total of 16 elements (K, P, Fe, Mg, Ca, Na, Cr, Zn, Ba, Mn, Ni, Cu, V, Sr, Cd and Co) were determined using an inductively coupled plasma atomic emission spectrophotometer (Shimadzu, Tokyo, Japan). Dry sample power (0.5 g) was accurately weighted with an electronic balance (Precisa, Zurich, Switzerland). Then they were transferred into the polytetrafluoroethylene (PTFE) pressure vessels and mixed with 6 mL HNO_3_ and 2 mL H_2_O_2_. This extract was digested with an automatic microwave digestion system. Finally, the digestion solution was filtrated and diluted to 25 mL using deionized water. Blank digestion was similarly prepared. Precision and accuracy were evaluated using standard reference material (GBW07605, Tea leaves). Differences between measured and certified values were all below 10%.

UV-Vis spectra were determined using an ultraviolet-visible spectrophotometer (Shimadzu, Tokyo, Japan). The scan range was set as 200–600 nm and the scan interval was set as 1 nm, respectively. The experimental conditions were based on a developed study by Li et al. [21]. Dry sample powder (0.1 g) was dissolved by 10 mL trichloromethane solution in a test tube for 20 min. Then the mixture was ultrasonically extracted at 150 W for 30 min and filtered by analytical filter paper. Extracted solution was used for the UV-Vis analysis.

FT-MIR spectra were determined using a Fourier transform mid-infrared spectrometer (Perkinelmer, Foster City, CA, USA). It was equipped with a deuterated tri-glycine sulfate detector. The scan range was 4000–400 cm^−1^ and the resolution was set as 4 cm^−1^, respectively. Each spectrum was recorded with 64 successive scans. The measurement of infrared spectra was based on a KBr pellet method. Each sample (1.0 mg) was evenly mixed with KBr (100.0 mg). Then they were pressed into a pellet by a tablet press (Shanghai Shanyue instrument Inc., Shanghai, China) for FT-MIR analysis. The pressure was set as 10 MPa. A blank KBr pellet was also recorded to remove the influence caused by the H_2_O and CO_2_ in the air.

### 2.3. Data Analysis

Sample characteristics were firstly explored based on each sensor system, respectively. A one-way analysis of variance was used to analyze each element with regard to two parts at a level of *P* ≤ 0.05. For the UV-Vis and FT-MIR datasets, an exploratory PCA that was widely used as a characterization tool was performed to present the distribution trend, respectively [22,23]. The loading plot was extracted to show the meaningful bands for exploratory analysis.

Subsequently, three data matrixes were fused according to a feature extraction procedure which can eliminate the colinearity and simplify the enormous dataset. The PLS-DA method was chosen to extract feature variables which are defined as latent variables (LVs) according to the lowest root mean square error of cross validation (RMSECV). This parameter can guarantee that feature variables are collected as much as possible and they are not overfitted. Finally, LVs extracted from each dataset were concentrated into a new data array for final origin traceability. In the process of feature extraction, variable importance in the projection (VIP) was applied to search for meaningful chemical information from each dataset to explain geographical difference from a chemometric point of view.

Based on fused data array, two supervised chemometric techniques were used for the origin traceability of *B. edulis* mushrooms. The PLS-DA used in our study is based on partial least square regression (PLS 1). It searches for related variables which have a maximum covariance with the “**Y**” response from the “**X**” data matrix. This method is a binary classification algorithm between zero and one [24]. A sample with **Y** = 1 indicates it belongs to a given class, while a sample with **Y** = 0 indicates it does not belong to this group. For example, a “**Y**” encoded {0; 0; 1; 0; 0; 0; 0; 0; 0; 0; 0} denotes that this sample belongs to the third class (Baohe, Weixi, Diqing). By continuous variable models, a predicted value will not be outputted as either one or zero perfectly, but it can estimate the probability of each sample being assigned to each group. According to published studies, a predicted value with a range from 0.5 to 1.5 was defined as the correct one [25,26,27,28].

Two parameters are important to evaluate the performance of a PLS-DA model. Root mean square error of estimation (RMSEE) is used to evaluate the calibration error [28]. The RMSECV is computed based on a cross-validation procedure (seven-fold). This parameter can effectively estimate the prediction ability of a classification model for unknown samples. These parameters are also related to the generalization ability of a PLS-DA model.

The other classification method, called grid search support vector machine (GS-SVM), was used to verify the PLS-DA result. Generally, it has a good generalization ability because it can better solve non-linear cases with the aid of kernels [29]. This method can map original data into a high dimensional feature space using a kernel, and an optimal hyperplane is created for an excellent separation [30]. Radial basis function was chosen because it can reduce the complexity of training data and therefore produce an excellent classification result [31]. With respect to this kernel, penalty parameter (C) and regularization parameter (γ) are two important parameters to determine the performance of a GS-SVM model. C is used to confirm the tradeoff between minimizing the training error and minimizing model complexity [32]. This value is closely related to the fitting degree and generalization ability of the classification model. γ is used to determine the non-linear feature hyperplane [32]. These parameters are selected according to the highest cross-validation (10-fold) based on a grid search method.

For the PLS-DA and GS-SVM methods, when calibrated models are developed according to their respective rules, two validation schemes are performed in sequence. The first one is called internal cross-validation and second one is called external validation for unknown samples. The former was evaluated by the accuracy of the calibration set. With respect to unknown samples, a classical kennard-stone algorithm was used to choose 1/3 samples as the validation set. The accuracy of the validation set was used to estimate the predicted ability of a classification model.

### 2.4. Software

The data analysis and chemometric models were performed using Simca (Version 13.0, Umetrics, Umeå, Sweden) and Matlab (Version R2017a, Mathworks, Natick, MA, USA). Omnic (Version 8.2, Thermo Fisher, Waltham, MA, USA) and UV Probe (Version 2.34, Shimadzu, Tokyo, Japan) were applied to treat the FT-MIR and UV-Vis spectral data.

## 3. Results and Discussion

### 3.1. Comparison Analysis between Cap and Stipe

#### 3.1.1. ICP-AES

**Figure 1:**
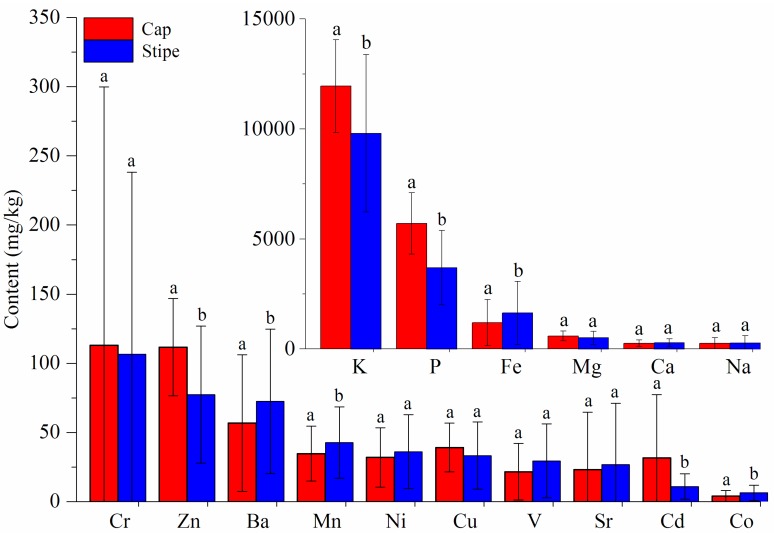
Comparison of 16 elements between cap and stipe. (Note: Different letters indicate a significant difference at *P* ≤ 0.05 according to Duncan test.)

The comparison of the 16 elements in the samples is shown in Figure 1. Elements of K, P, Fe, Mg, Ca, Na, Cr and Zn (exceeding 100 mg/kg) are more-highly accumulated than others. Accumulations of K, P, Fe, Zn, Ba, Mn, Cd and Co between cap and stipe show significant variations (*P* ≤ 0.05) indicating that these elements change severely depending on different parts. Higher levels of K, P, Zn and Cd are accumulated in the cap while Fe, Ba, Mn and Co are more concentrated in the stipe. Other elements (Mg, Ca, Na, Cr, Ni, Cu, V and Sr) have no significant variations between the cap and stipe. Additionally, it is noteworthy that the standard deviations of Na, Cr, Sr and Co are very large. These obvious intragroup variations may be mainly caused by different geographical origins. Many previous studies [33,34,35,36] have proved that soil condition and human activities in a certain geographical origin strongly affect the level of elements in mushrooms.

#### 3.1.2. UV-Vis

**Figure 2:**
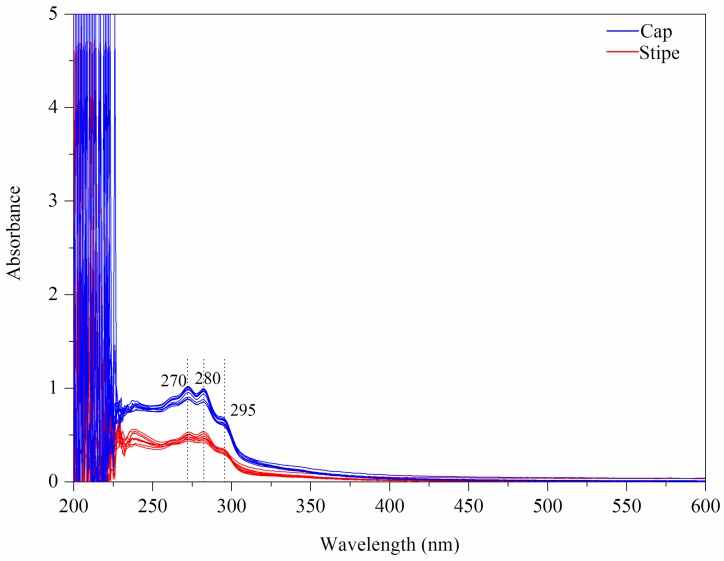
UV-Vis fingerprints of mushroom samples.

**Figure 3:**
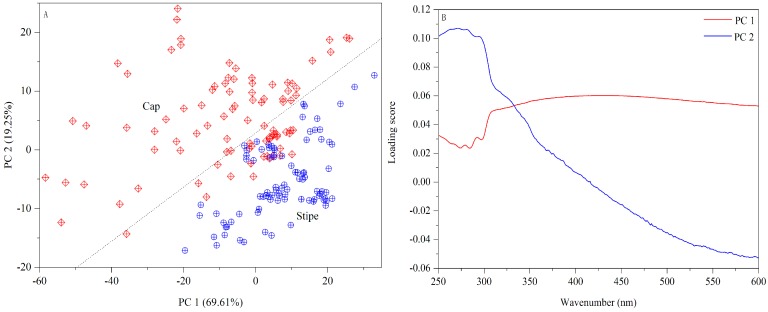
PCA result based on UV-Vis spectra ((**A**) Score plot; (**B**) Loading plot).

The UV-Vis fingerprints of mushroom chloroform extract (Wenshui, Bajie, Anning, Kunming, China) are presented in Figure 2. Except solvent peaks, the main absorptions are observed at 270, 280 and 295 nm in the UV region and no absorptions are emerged in the Vis region. The band of 280 nm may arise due to protein compounds [37]. With respect to 295 nm, this peak may be attributed to phenolic constitutes which are closely related to the antioxidant properties of mushrooms [38,39]. Visually, the spectral difference between cap and stipe is obvious in terms of absorptive intensity. According to previous studies [40,41], the mushroom cap has more protein accumulation and higher antioxidant capacity than the stipe. A PCA score plot is shown in Figure 3A. Some overlapped samples are presented indicating that some interference may be introduced due to different sampling locations. The loading plot indicates that bands of 330–600 nm make a greater contribution to the first principle component (PC) and that 250–330 nm bands make a greater contribution to PC 2 (Figure 3B). The UV-Vis fingerprints of mushroom chloroform extract from other geographical origins are shown in Figure S3.

#### 3.1.3. FT-MIR

**Figure 4:**
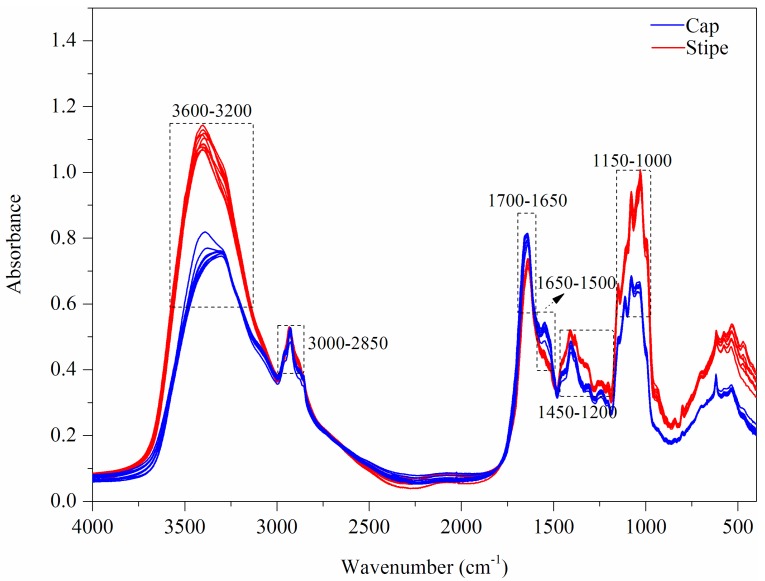
FT-MIR fingerprints of mushroom samples.

The FT-MIR spectra of mushroom samples (Wenshui, Bajie, Anning, Kunming, China) are displayed in Figure 4. Broad bands of 3600–3200 cm^−1^ are attributed to hydroxyl stretch absorption. This region is always not chosen for chemometric analysis because it is mostly caused by intense water interference. The 3000–2850 cm^−1^ bands may belong to the methylene group dominated by fatty acid compounds [42]. With respect to 1700–1500 cm^−1^, this portion is closely related to protein constitutes. Peaks around 1655 and 1550 cm^−1^ are mainly caused by amide І and amide П ingredients, respectively [43]. The 1450–1200 cm^−1^ bands are complicated because many small peaks are raised in this region. A study for the discrimination of seven porcini species assigned these peaks as mixed vibration absorptions of protein, fatty acids and polysaccharides [44]. Mohaček-Grošev et al. [45] have indicated that some peaks such as 1460, 1410, 1380, 1316, 1260, 1202 cm^−1^ may be the result of a pyranose ring structure. In addition, the band of 1000–1200 cm^−1^ is an obvious carbohydrate area that has two sharp peaks of 1032 and 1080 cm^−1^ that are mainly assigned as the structure of chitin [46,47]. Chitin is a major structural polysaccharide in macrofungi [47]. The 900–800 cm^−1^ region may be assigned as glucan and mannan bands [45]. The rest bands below 900 cm^−1^ are the fingerprint region where many peaks are unidentified. However, many studies have showed that this region may be useful for a good chemometric analysis.

**Figure 5:**
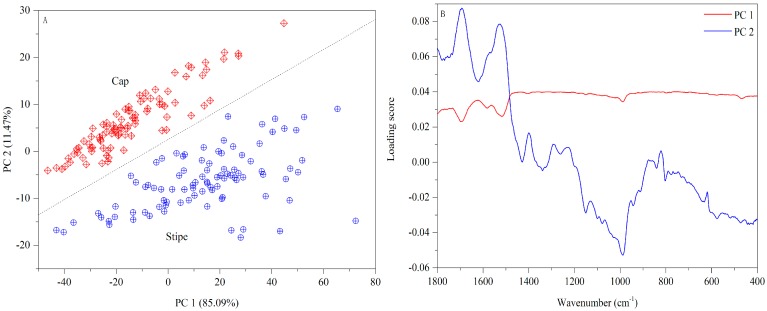
PCA result based on FT-MIR spectra ((**A**) Score plot; (**B**) Loading plot).

A PCA score plot based on FT-MIR spectra is exhibited in Figure 5A. Visually, the first two PCs can provide an excellent separation between two parts. The loading plot (Figure 5B) shows that 1500–400 cm^−1^ bands make a higher contribution to PC 1 while 1800–1500 cm^−1^ bands are more important to PC 2. The FT-MIR fingerprints of the mushroom samples from other geographical origins are shown in Figure S4.

### 3.2. Regional Differences Based on VIP

#### 3.2.1. ICP-AES Dataset

**Figure 6:**
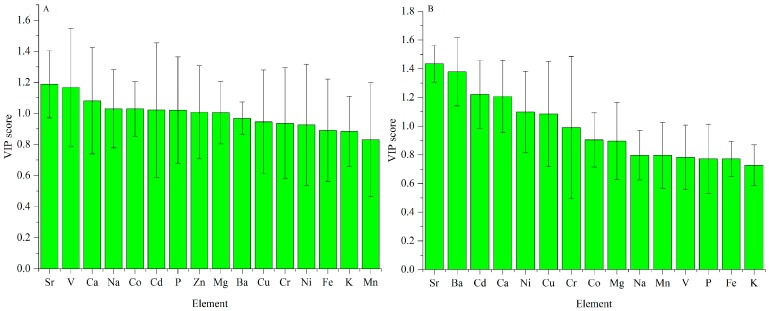
VIP scores of ICP-AES data for regional difference ((**A**) Cap; (**B**) stipe).

For the cap data, the VIP scores are exhibited in Figure 6A. Sr and V are the most significant elements for explaining the variation caused by geographical origins. Scores of Ca, Na, Co, Cd, P, Zn and Mg are higher than one, showing that they are also important.

Similarly, the VIP scores for the stipe data are exhibited in Figure 6B. Sr and Ba are the most important elements for geographical difference. Elements of Cd, Ca, Ni and Cu also make a greater contribution because their VIP values are higher than one.

Sr is the most important element for explaining the regional difference both in the cap and stipe, which is consistent with the high standard deviation in Figure 1. Similar conclusions were also concluded according to previous chemometric analyses for elements in *B. edulis*. Sr, Na, V, Ca, Ni and Ba were proven to be important elements for discriminating fruiting bodies of this mushroom from different geographical origins [48]. Based on PCA loading analysis, elements of Sr, Al, Ba, Ca, Fe and Mn in the cap, and Ca, Sr, Ba, Al, Mn and Fe in the stipe have a strong influence on geographical variation (11 different sites across Poland) of King Bolete [49]. However, this conclusion may be species-independent. For *Pleurotus tuber-regium*, elements of Co, Cr, Fe, Hg, Na, Ni, and Pb are important variables for interpreting their variation based on two sites in Nigeria [50]. Another study on *Sarcodon imbricatus* indicated that elements of Ag, As, Cd, Cu, Cs, etc. make a great contribution to the PCA exploratory analysis [51].

#### 3.2.2. UV-Vis Dataset

**Figure 7:**
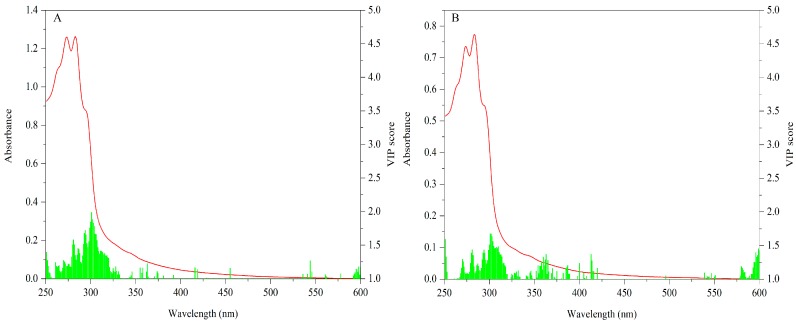
VIP scores of UV-Vis data for regional difference ((**A**) Cap; (**B**) stipe).

The VIP scores (values ≥ 1) of cap data are shown in Figure 7A. Significant variables are mainly concentrated in 260–330 nm. Especially absorptions around 295 nm make the most contribution. With respect to the stipe, VIP scores (values ≥ 1) for this data matrix are shown in Figure 7B. Similarly, 260–330 nm is the main contributive region and the peak around 295 nm is the most important variable.

The important bands of both the cap and stipe for explaining geographical variation are similar. Different levels of some characteristic compounds such as protein and phenols may be responsible for this difference. Additionally, because spectra absorption mainly arose in the UV region, importance variables mostly appeared in this area. The UV region has been successfully used to describe variation among mushrooms. Li et al. [52] have proven the feasibility of this region for discriminating *Wolfiporia extensa* (a Chinese traditional mushroom) from different geographical origins. The UV region was also successfully applied to classify different bolete species, showing that 270–300 nm bands make the most important contribution [21].

#### 3.2.3. FT-MIR Dataset

**Figure 8:**
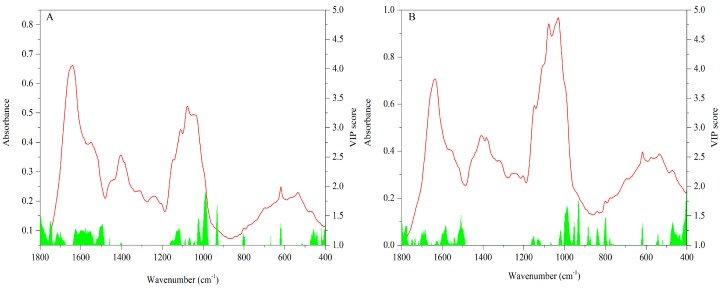
VIP scores of FT-MIR data for regional difference ((**A**) Cap; (**B**) stipe).

Compared with the above sensory techniques, the FT-MIR technique can present abundant and complicated descriptive information for mushroom samples due to its high-throughput capacity. The VIP scores (values ≥ 1) of the cap data are shown in Figure 8A. The regions of 1800–1500 and 1200–900 cm^−1^ make a great contribution to the geographical difference. These characteristic bands are mainly assigned to protein and polysaccharide compounds, respectively. Some non-identified compounds in the fingerprint region are also important.

For the stipe samples, VIP scores (values ≥ 1) are shown in Figure 8B. The contributive bands are very similar to those of the cap data that 1800–1500, 1200–900 cm^−1^ and some peaks in the fingerprint region generate an important contribution for geographical difference.

The protein and polysaccharide bands make an important contribution to geographical difference. Many studies have demonstrated that these constituent elements are prominent in the mushroom fruiting body and they are always varied on the basis of different geographical origins [53]. In our study, this conclusion is proved from the chemometric point of view by using FT-MIR spectra.

### 3.3. Origin Traceability Based on Chemometrics

#### 3.3.1. Cap

A total of 56 variables containing 11 LVs from ICP-AES, 16 LVs from UV-Vis and 29 LVs from FT-MIR data matrixes were used to develop PLS-DA and GS-SVM models, respectively. The results are shown in Table 2 and Table 3. With respect to PLS-DA, the accuracies for the calibration set and validation set were 100% and 90.625%, respectively. Two samples from Diqing city and one sample from Dali city were incorrectly predicted. The generalization ability was also acceptable, with a low RMSEE and RMSECV of 0.076 and 0.251, respectively. Comparatively, a better result was obtained using GS-SVM methods. Accuracies for both the calibration set and validation set are 100% and the parameters of C and γ were 1 and 0.044194, respectively (Table 3). Score plots of the GS-SVM model are shown in Figure S5.

#### 3.3.2. Stipe

**Figure 9:**
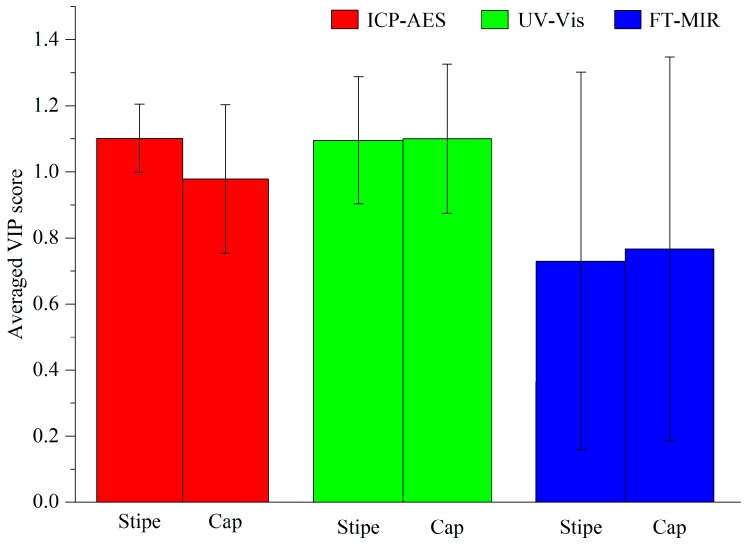
Averaged VIP score of each data matrix between cap and stipe models.

Similarly, eight LVs from ICP-AES, 16 LVs from UV-Vis and 30 LVs from FT-MIR were used to concentrate into a new data array. Based on the PLS-DA technique, 100% and 96.875% accuracies with respect to the calibration set and validation set were calculated, respectively. Only one sample from Diqing city was incorrectly predicted. The RMSEE and RMSECV were 0.079 and 0.244, respectively (Table 2). Comparatively, according to this method, the generalization ability using the stipe data may be better than that using cap data for geographical characterization, with a higher prediction accuracy and a lower RMSECV. In order to understand which dataset led to this result, the averaged VIP score of each data matrix for the cap and stipe models were extracted, shown in Figure 9. As can be seen in this figure, the contribution of the ICP-AES data to the stipe model is obviously higher than that for the cap model. The result proves that the accumulation of the elements in the mushroom stipe may have a stronger correlation to geographical origins than that in the mushroom cap. This result may be caused by the stronger relationship of the stipe than that of the cap.

Additionally, the discrimination performance of GS-SVM was better than that of the PLS-DA method due its excellent capacity for non-linear conditions. The 100% accuracies with regard to the calibration set and unknown samples were calculated with a small C and γ of 1 and 0.0625, respectively. Score plots for the GS-SVM model are shown in Figure S6.

Conclusively, the origin traceability of *B. edulis* mushrooms can be successfully discriminated based on either the cap or stipe data, in combination with the PLS-DA and GS-SVM methods. The accuracies of these models range from 90.625% to 100% and their errors are minor, showing that these models also have a good generalization ability. Comparatively, the cap may have a better potential for the origin traceability of *B. edulis* mushrooms. Different parts of porcini mushrooms (pileipellis, flesh and hymenium) and *Wolfiporia extensa* (the epidermis and inner part) have been used for chemometric characterization of different mushroom samples, exhibiting that the classification potential among different parts is different [54,55]. With respect to two chemometric techniques, the performance of GS-SVM is always better than PLS-DA. This could be contributed to its capacity for solving non-linear problems with the help of kernels. The relationship between sensory data and geographical labels may be complicated and non-linear. Therefore, a non-linear classification technique may be superior to a linear one for the origin traceability of this species.

## 4. Conclusions

Three sensor systems—ICP-AES, UV-Vis and FT-MIR—were applied for the origin traceability of *B. edulis* mushrooms (cap and stipe) from 11 collection origins. The chemical variation between the cap and stipe was first analyzed based on a single sensor system. Sr, V, Ba, protein and polysaccharide constitutes were proven to be important variables for regional variation.

Feature variables from three data matrixes were concentrated into a new data array. PLS-DA and GS-SVM were used as chemometric methods to develop mathematical models with regard to the cap and stipe, respectively. The discrimination performance was satisfactory with accuracies ranging from 90.625% to 100%. These models also have an excellent generalization ability with reliable evaluation parameters. Our study has showed that a multi-source data fusion strategy can be successfully applied to characterize *B. edulis* mushrooms (caps and stipes) on the basis of different geographical regions.

## Figures and Tables

**Table 1:** Information of *B. edulis* samples.

Geographical Origins	Quantity	Longitude	Latitude	Altitude (m)
Potatso national park, Xianggelila, Diqing	10	99.908	27.802	3515
Midu, Dali	10	100.491	25.344	1670
Baohe, Weixi, Diqing	7	99.286	27.177	2300
Longyang, Baoshan	10	99.166	25.121	1680
Wenshui, Bajie, Anning, Kunming	9	102.393	24.577	1846
Fengyi, Bajie, Anning, Kunming	10	102.333	24.692	1984
Tongchang, Yimen, Yuxi	7	102.039	24.714	2198
Songgui, Heqing, Dali	6	100.210	26.354	1944
Dongshan, Wenshan	6	104.281	23.400	1430
Liujie, Bajie, Anning, Kunming	8	102.686	24.532	1998
Suoyishan, Weize, Shilin, Kunming	9	103.346	24.645	1893

**Table 2:** Results of PLS-DA models.

Model	RMSEE	RMSECV	Accuracy of Calibration Set	Accuracy of Validation Set
Cap	0.076	0.251	100.000%	90.625%
Stipe	0.079	0.244	100.000%	96.875%

**Table 3:** Results of GS-SVM models.

Model	C	γ	Accuracy of Calibration Set	Accuracy of Test Set
Cap	1.000	0.044194	100.000%	100.000%
Stipe	1.000	0.0625	100.000%	100.000%

## Supplementary Materials

The following are available online at www.mdpi.com/1424-8220/18/1/241/s1, Figure S1: Eleven microhabitats of *Boletus edulis* in Yunnan Province, Figure S2: The real product of *Boletus edulis*, Figure S3: The UV-Vis fingerprints of mushroom chloroform extract from other ten geographical origins, Figure S4: The FT-MIR fingerprints of the mushroom samples from other ten geographical origins, Figure S5: Score plots of the GS-SVM model of mushroom cap, Figure S6: Score plots of the GS-SVM model of mushroom stipe.
