# Supplementary material for: Feature Fusion of ICP-AES, UV-Vis and FT-MIR for Origin Traceability of Boletus edulis Mushrooms in Combination with Chemometrics
Source: Sensors (Basel). 2018 Jan 15;18(1):241. doi: 10.3390/s18010241 (PMC5795700; doi:10.3390/s18010241)
Supplement: Supplementary file 1 [file sensors-18-00241-s001.pdf]

Article

Feature Fusion of ICP-AES, UV-Vis and FT-MIR for Origin Traceability of *Boletus Edulis* Mushrooms in Combination with Chemometrics

Luming Qi [1,2], Honggao Liu [3], Jieqing Li [3], Tao Li [4,*] and Yuanzhong Wang [1,*]

1 Institute of Medicinal Plants, Yunnan Academy of Agricultural Sciences, Kunming 650200, China; 18669326801@163.com
2 State Key Laboratory Breeding Base of Systematic Research, Development and Utilization of Chinese Medicine Resources, Chengdu University of Traditional Chinese Medicine, Chengdu 611137, China
3 College of Agronomy and Biotechnology, Yunnan Agricultural University, Kunming 650201, China; honggaoliu@126.com (H.L.); lijieqing2008@126.com (J.L.)
4 College of Resources and Environment, Yuxi Normal University, Yuxi 653100, China
* Correspondence: ltyx_1976@126.com (T.L.); boletus@126.com (Y.W.)

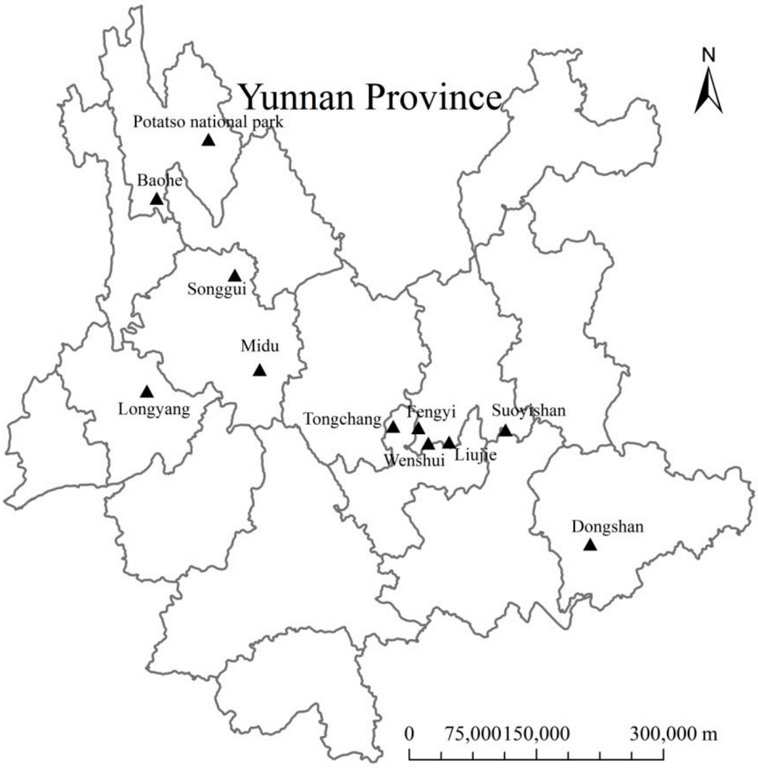

Figure S1: Eleven microhabitats of *Boletus edulis* in Yunnan Province

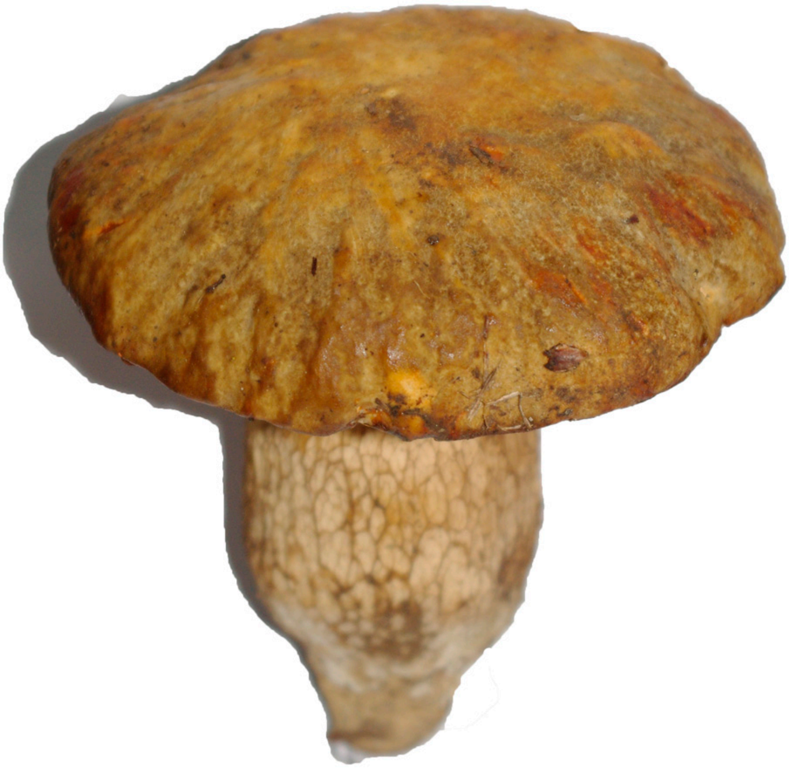

Figure S2: The real product of *Boletus edulis*

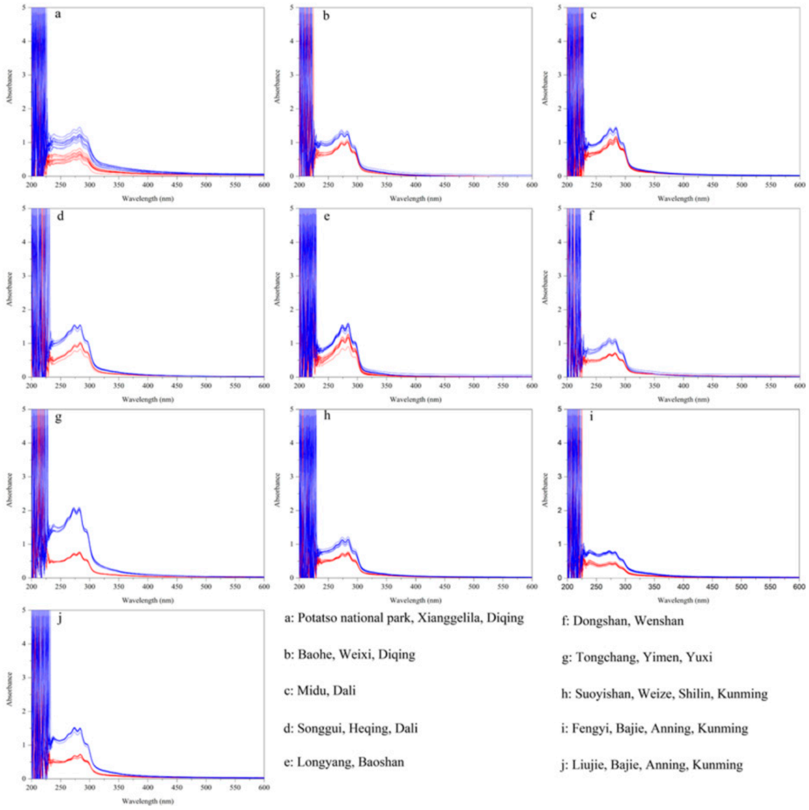

Figure S3: The UV-Vis fingerprints of mushroom chloroform extract from other ten geographical origins

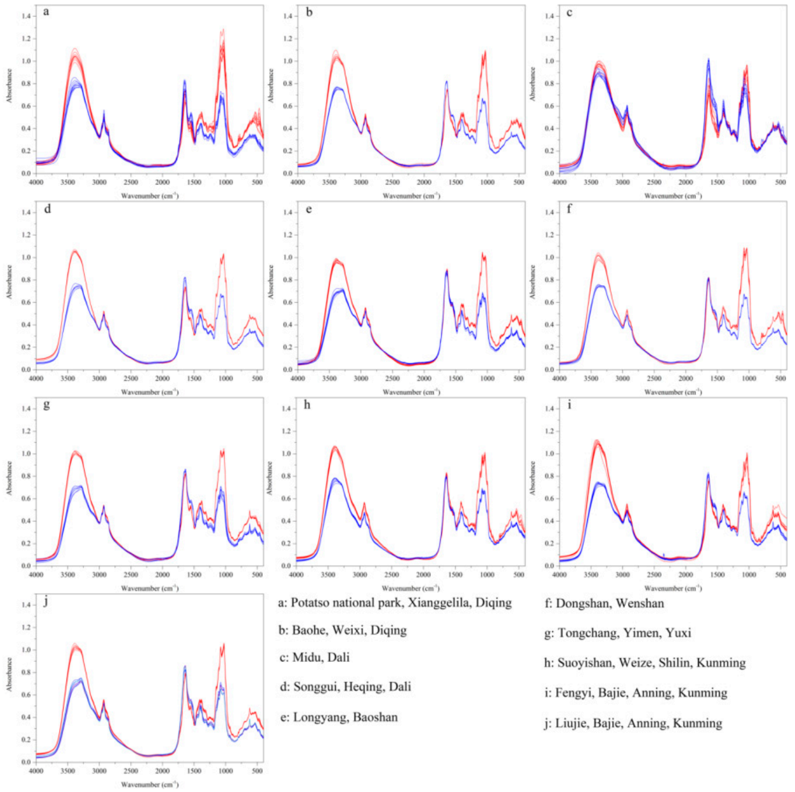

Figure S4: The FT-MIR fingerprints of the mushroom samples from other ten geographical origins

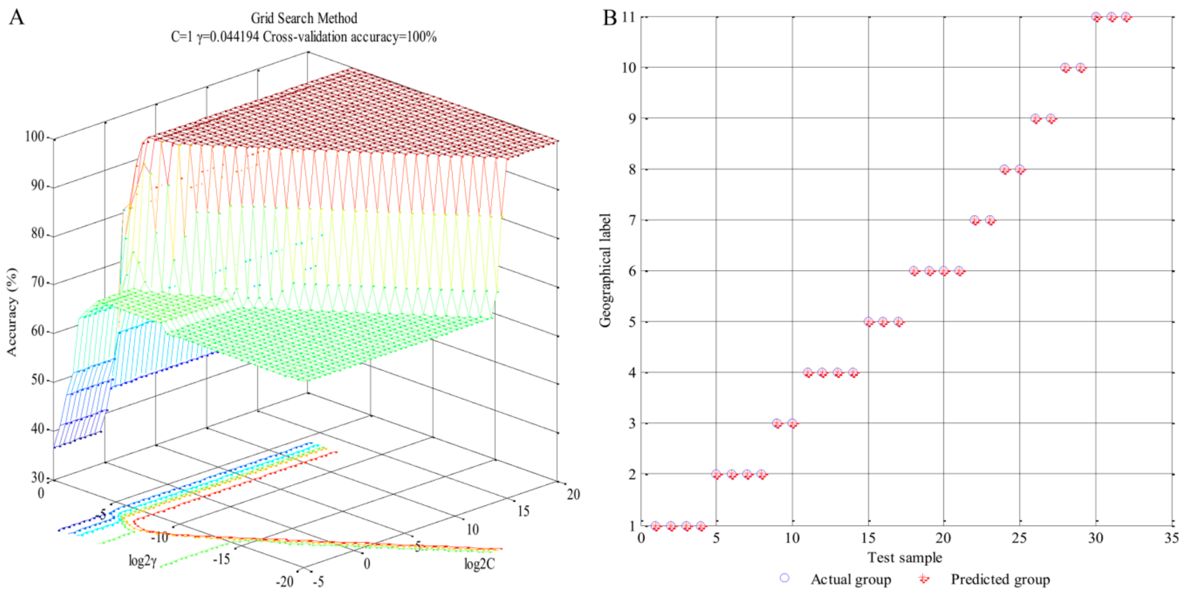

Figure S5: Score plots of the GS-SVM model of mushroom cap

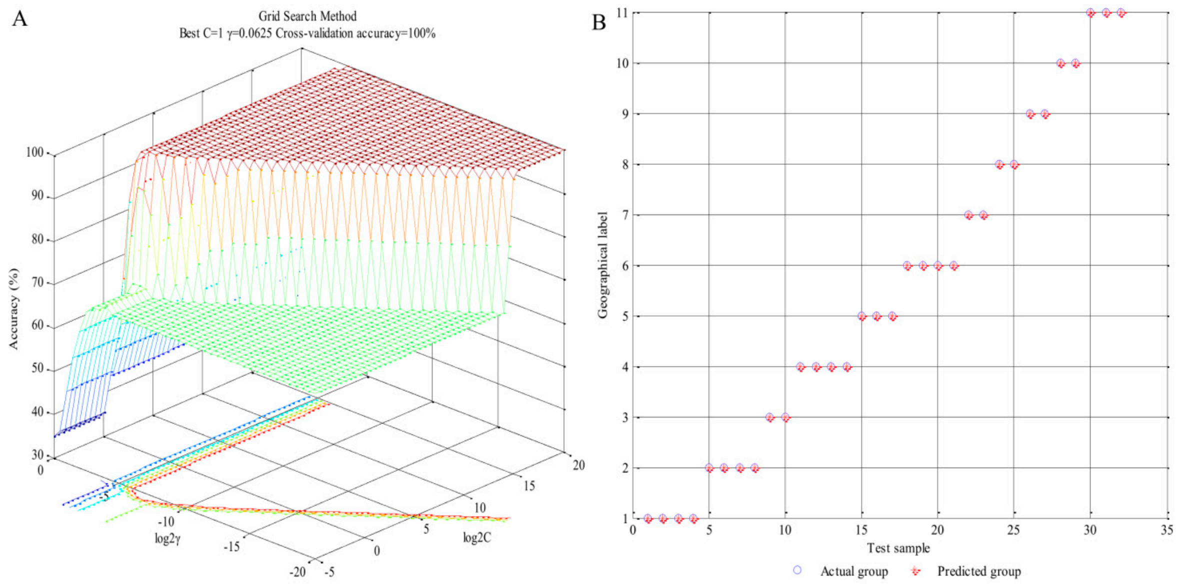

Figure S6: Score plots of the GS-SVM model of mushroom stipe
